# The barriers to receiving health care for people with Parkinson’s from predominantly Asian backgrounds in the UK

**DOI:** 10.1038/s41531-025-00946-9

**Published:** 2025-05-20

**Authors:** Mouhammed Ramadhan, Joshua Stott, Anette Schrag

**Affiliations:** 1 Department of Clinical and Movement Neurosciences, UCL Queen Square Institute of Neurology, University College London, London, UK; 2 Division of Psychology and Language Sciences, University College London, London, UK

**Keywords:** Parkinson's disease, Parkinson's disease

## Abstract

Despite universal access to healthcare to eligible people of all ethnic backgrounds in the NHS, disparity in healthcare provision exists. Parkinson’s disease (PD) is a chronic, progressive disorder with an insidious onset and increasing healthcare needs over time. However, there is little information on the experience of healthcare and barriers to access in people from minority ethnic (ME) backgrounds in the UK. Interviews were conducted with 21 People with Parkinson’s (PwP) (38% female, mean age 54 (SD 10.67 years) from ME groups. Results were analysed using Braun and Clarke’s thematic analysis. Four main themes were extracted: Awareness and Acceptance of Symptoms in the ME Community; Socio-cultural Expectations and Impacts; Access to Information on Parkinson’s and Services; and Experiences of Healthcare. ME-PwP experience challenges in accessing healthcare, even in a universal healthcare system. Recognising these complex barriers may improve access and quality of life.

## Introduction

Despite universal access to healthcare for eligible people of all ethnic backgrounds in the NHS, disparity in healthcare provision exist, with people from minority ethnic (ME) groups known to access fewer healthcare resources^1^. Parkinson’s disease (PD) is a chronic, progressive disorder with an insidious onset and increasing healthcare needs over time. Despite the growing recognition of PD’s impact across diverse communities^1,2^, very little information exists on the experience of healthcare in people with Parkinson’s (PwP) from ME groups and the barriers they face in accessing healthcare even in a universal healthcare system such as the NHS. Existing research has predominantly centred on American Black, African American, and Hispanic populations in the United States. There is evidence suggesting that people from ME groups have lower rates in early PD diagnosis and treatment to clinical trial participation, which affects health outcomes and limits generalisable data^3^. ME-PwP show lower clinical engagement, marked by high rates of missed appointments and cancellations^4^. It has been reported that they encounter unique challenges in healthcare accessibility, influenced by a complex interplay of health literacy, linguistic, cultural and systemic factors^5^. Such challenges can not only exacerbate disease burden but have also been shown to result in decreased access to preventive care and treatment for chronic conditions which leads to worse health outcomes and an increased likelihood of developing other serious illnesses^6^. We here report the experiences of PwP from EMs in order to identify the barriers to healthcare access to help improve their care and healthcare equity. While we recognise that the term Minority Ethnic encompasses hugely diverse populations, there are some commonalities in their experience, and we know, for example, that ME groups from all backgrounds experience delayed diagnosis^7,8^. Given the scarcity of knowledge in this area we sought to understand commonalities, pointing to within-group diversity where appropriate.

## Results

**Fig. 1:**
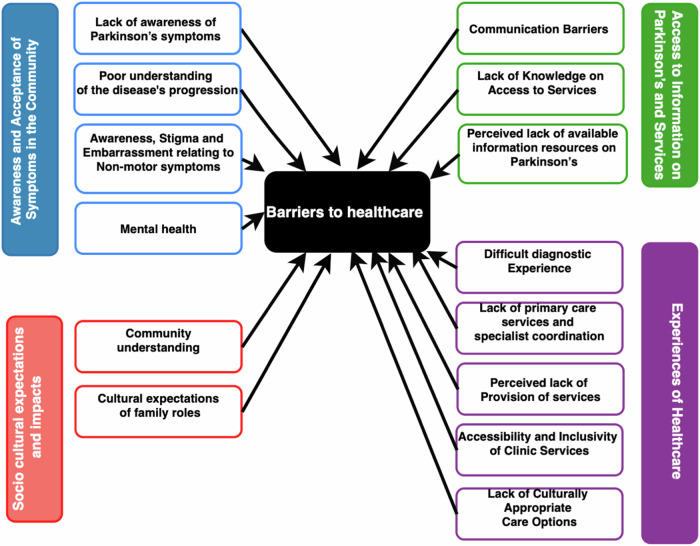
Barriers to receiving healthcare for Minority Ethnic groups with Parkinson’s.

The study identified four key themes: ‘Awareness and Acceptance of Symptoms in the ME Community’, ‘Socio cultural Expectations and Impacts,’ Access to Information on Parkinson’s and Services’, and ‘Experiences of Healthcare’ (Supplementary Table 3). The themes all intersected around barriers to healthcare and this is illustrated in Fig. 1.

### Awareness and acceptance of symptoms in the minority ethnic community

Lack of awareness of Parkinson’s symptoms was reported by the majority of participants within ethnic minority communities, often leading to delays in healthcare seeking. While most recognized the overt physical features such as tremor, the other manifestations of the disease such as non-motor symptoms were underappreciated.

*“So culturally, I don’t think many people know what Parkinson’s is or if they do, they only think about tremors, handshakes and that’s it. They don’t realise all the other things that go on in your body, in your head and the lack of sleep and the tiredness, the fatigue people don’t understand that at all.” (P13)*

Due to the lack of awareness some attributed Parkinson’s symptoms to other, usually more benign, causes leading to delays in diagnosis (*“he thought that he had some deficiency in like vitamin deficiency and over burden of work”)*. For some this lack of awareness, compounded by a potential reluctance to accept the reality of the disease, created a barrier:

*“ When it comes to the reality it was a bit hard to accept for some time.” (P3)*

For some poor understanding of the disease’s progression led to emotional distress and confusion in response to the onset of new symptoms. There was an apparent lack of understanding or acceptance of why these symptoms are emerging, suggesting a possible unawareness of the disease’s progression and possible symptoms.

Other participants expressed dissatisfaction and concern about the inefficacy of the prescribed medication, potentially reflecting lack of awareness of the progressive nature of Parkinson’s or expected medication responses:

*“Two or three days I’ll be angry, I’ll be upset, I’ll be depressed and because it’s like a new feeling, why I’m having this problem?” (P4)*

*“Yeah, I think you know it is a lot of uncertainty about Parkinson’s because I’m taking tablets and I feel I’m getting worse.” (P7)*

*“My neurologist did start me on Sinemet which she thought might help me, but it hasn’t changed anything that about the way that I feel. It’s only 5 milligrams. But it hasn’t. I’ve been on it for about 6/7 weeks now and it hasn’t made any difference yet.” (P2)*

Stigma and Embarrassment relating to Non-Motor Symptoms was evident in some cultures. Impairment of motor skills or balance appeared more recognised and accepted as features of PD (“*Yes, his tremor is not that bad? His balance is awful”)* than other symptoms such as non-motor symptoms which were often not associated with PD, *“Non motor symptoms would you explain what you mean by non-motor symptoms”*. (P11)

For many participants stigma and shame were barriers that limited open communication making many participants reluctant to discuss intimate non-motor issues unless directly questioned, often responding hesitantly. This reluctance was especially pronounced among Muslim participants, regardless of their South Asian or Arab backgrounds, but was also observed among individuals from other religious backgrounds, including Christians, Hindus, and Sikhs from South Asia. Embarrassment over non-motor symptoms, such as urinary problems, often prevented individuals from discussing these issues and became a barrier to attending clinics (“*Those symptoms that are embarrassing, like urinary problems. Uh. Yeah, this kind of things*”).

*“It is to do Pakistani culture; he feels that that he is shy and reluctant to discuss.” (P11)*

Participants demonstrated a reluctance to discuss embarrassing symptoms of Parkinson’s also with family members:

*“It is usually incontinence, and like that […] “Urine control and constipation.” (P11)*

*“I usually go with my husband. To the doctor. So, it’s quite embarrassing to talk about it.” (P5)*

*“Yeah, I think sexual health is something that a lot of Asian people would have difficulty discussing. I personally think because I’m from a medical background I just get on with it.” (P2)*

Participants highlighted the deeply personal nature of non-motor symptoms and how people with PD might feel compelled to hide them. When asked which symptoms are the most difficult to discuss, participants often mentioned “*incontinence and constipation, this kind of symptoms*”. The desire to shield oneself from potential judgement or misunderstanding reflects fear of societal and family reactions (stigma):

*“Yes, definitely because they’re more personal, so therefore you try and keep quiet about it, you know,” (P13)*

*“When I started to have those urine urinary problems, I wrote it. It coincided with the COVID, […] I didn’t have to go to the doctor. But if we were not in lockdown. I would have done it the same way I would have written to the doctor by e-mail, and she responded to me by e-mail.” (P5)*

Mental health concerns, such as anger, depression and anxiety were often viewed differently from physical symptoms. The perceived stigma around mental health in their communities led to particular reluctance in discussing these issues. This strong mental health stigma compounded existing barriers to seeking healthcare, as some expressed fear of being perceived as weak.

*“ I don’t want anyone to say that ohh she is struggling, you know? So to be honest I try to hide a lot.” (P3)*

*“I felt embarrassed at the beginning […] as if I was weak.” (P5)*

### Socio-cultural expectations and impacts

A lack of understanding of Parkinson’s within the wider community was frequently mentioned:

*“People don’t know about the Parkinson’s. They don’t know about the Parkinson’s. They don’t know about that it is mental condition or neurological condition. They see your body features.” (P11)*

*“Less awareness that’s obvious, even people who you live with are unaware so how would the others be aware.” (P6)*

*“Sometimes they say, why you feeling cold when she’s got tremble, you know, are you feeling cold? Oh. Let me get you a blanket, you know, don’t understand it is part of Parkinson’s.” (P13)*

Cultural expectations of family roles placed additional pressures, particularly on South Asian women. Some reflected on the internalized cultural values that emphasize family over personal well-being. This expectation often lead to self-inflicted pressure to prioritize familial responsibilities above their own needs, particularly when it came to seeking medical care for Parkinson’s symptoms.

*“I think it’s a cultural trait amongst South Asian women that we don’t always put ourselves first, and we have a tendency to try put on a brave face and crack on […] So I think there was very much that Asian mentality that you’ve got to be strong […]when I used to see I had these symptoms developing so my idea was due to cultural. Yeah, I suppose is that we don’t get unwell, we just crack on.” (P2)*

### Access to Information on Parkinson’s and services

Some highlighted linguistic challenges, creating a barrier in understanding and accessing information and healthcare. Other participants also felt being from an ethnic or cultural background affected how they communicate their health problems:

*“I’m typical of a second generation Asian and that I don’t want to make a fuss. We are always taught from a young age we’re a guest in this country so don’t make a fuss. You know, like standing in the line properly, don’t rattle the feathers. [..] Whereas the people who aren’t second generation immigrants from South Asian origin might make a fuss, in fact they do make a fuss. That’s why they get what they want”. (P2)*

A lack of knowledge on how to access Parkinson’s services led to feelings of isolation among the majority of participants who felt they did not know where or how to seek help.

*“But I think a lot, many, people especially in our community might not know how to access services and resources, and I think that’s a big issue. You know, because nobody told us how to access and everything. We just, we just, you know, have to learn ourselves.” (P13)*

A perceived lack of available information resources on Parkinson’s left participants feeling uncertain about how to manage their condition, as they did not have clear guidance on PD care and available support.

*“No. No. Not at all. Not available here. Even they don’t give you the guidelines what to do you have to care for and how to.” (P11)*

*“When my wife was diagnosed, and we weren’t given anything in writing. It was just like, you know, here the medications and go and get on with it. But I think we should, we need more”. (P13)*

### Experiences of healthcare

Some participants were reluctant to seek help due to fear of stigmatisation and judgement when considering visiting a GP. The fear of misunderstanding or stigmatization could be a barrier to early diagnosis:

*“I was thinking if I go with this to the GP then she might be thinking oh why did she come here for the this? ... That was my initial thought.” (P2)*

In others there was a perceived dismissiveness from healthcare providers as they felt unheard despite explicitly communicating their symptoms. This diminished their trust in healthcare systems and prevented future visits:

*“No, I told him about this. I told everybody. I told him about it, but they didn’t seem to convince them, or didn’t seem to make them believe it, or I don’t know, I don’t know.” (P6)*

Participants often described how medical practitioners misattributed or dismissed their symptoms *(“They were saying it’s an orthopaedic problem”)*. Some participants felt unheard and misunderstood suggesting that the problem was psychological and not physical*” (you just imagining, you know, you’re putting so much pressure that you can’t walk, and you think you can’t walk anymore?” (P15)*).

Some participants felt that a lack of understanding and empathy in their diagnostic experience led to the healthcare system initially dismissing the seriousness of their condition, causing delays in receiving the needed care *(“Yeah, because it wasn’t seen as serious at first”)*.

For some, they felt healthcare providers focused more on the diagnosis than the participant, making the process impersonal and uncomfortable for the individual after having a negative experience with the initial healthcare providers (GP’s) participants would be reluctant to visit future specialist neurologist clinics:

*“You know I went there on my own […] I was really shocked because he made it all about himself and his diagnosis, and there was no concern, or you know. For me it was all about him.” (P7)*

Some Participants felt that, due to lack of primary care services, some providers such as GPs are too busy to give them the attention and time needed (*“Because if you go to the doctors into the GP, they haven’t got time”)*. Others touched upon the lack of specialist coordination and sluggishness of the medical system, emphasizing the inefficiency and delay in service access (“*The delay was the medical part as in we weren’t getting appointments quickly enough”;* *“They don’t have appointments until two weeks”).* Others mentioned that access to specialist knowledge is an area that needs improving:

*“An area that honestly needs to be improved In my GP, there’s barely no one specialized in Parkinson or specialized in movement disorders in general […]I go to my neurologist who is specialized in Parkinson every six months and every six months is not sufficient at all for Parkinson because things can change quickly and you know I try to keep track of my symptoms on a on an Excel sheet and when I go to the neurologist” (P5)*.

Some participants felt there was a disconnect between different healthcare providers, leaving the person unsure about available services and treatment protocols:

*“Because there’s the neurologist saying one thing the psychiatrist says another thing the pharmacist says another thing about the levels of medication, what medication to give, what to use, what dosage to do, and they don’t seem to get together to talk to each other and we’re stuck in the middle.” (P13)*

*“I have a physiotherapist and occupational therapist. But it’s all the specialists there they’re all quite independent. You know they’re not really. It’s not really a collective connected service” (P1)*

*“But when it comes to communication between them and getting the heads together to try and tweak the medication, that’s when the problem is […] So we’ve got to, we’ve had to fight. We’ve had to push for these things, you know, and that’s and even to get the care for my wife is taken us a long time to get the extra care for her, you know. So people, if they don’t know who to access, who to, how to talk with them and what to do, then they will not get the care […] it’s tough, it’s tough, you know, so it’s taken us a few years to get here.” (P13)*

There was a perceived lack of service provision, as the majority of participants seemed unaware or were not supported by services for PwP like a PD nurse or a physiotherapist, and few perceived this as being less available for them than for other populations.

*“I don’t have a PD nurse. I don’t have a physiotherapist. I have a neurologist.” (P2)*

*“I think there they might be available, but we might not always be able to access them. There’s a difference. You know, I think so. The having extra care is available. Having a neurologist is available, but if you don’t know how to access these people or how to get them to communicate to each other, that’s the problem I think.” (P13)*

Overall, most participants felt that accessibility and inclusivity of clinic services were adequate, as they did not feel any racism or disparity in the treatment they received. The majority of participants did not feel ethnicity, religion or culture affected how they were treated, *(“I think we’ve been given good healthcare”) or some mentioned (“I don’t think I’m at a disadvantage from because of my ethnicity”), (“So far no. So far, I’ve never felt discriminated”), (“Well, it’s helping because we’ve got things out at our disposal, but we have to ask for them or to look for them or to yeah to ask about them, yeah”)*.

*“I don’t think it’s a cultural issue from that point of view or even the religious point. I think it’s more of a us understanding Parkinson’s and also to get the professionals to understand our needs […] I don’t think we get kind of less or more of a service because of because of the ethnic minority or because we are Muslims […] It’s just we could be anybody, […] in Bradford there was only one Parkinson’s nurse for a long time. Now we’ve got two and thus improve the services […] I think there’s only a few, maybe one or two neurologists dealing with all the Parkinson’s in Bradford you know. So, we could do with more professionals. […] That would help I think.” P13*

However, participants mentioned that there’s a noticeable lack of diversity in the healthcare workforce, pointing out the need for increased diversity to better serve diverse communities.

*“But I myself haven’t had any issues, but I don’t know many neurologists who are Asian.” (P2)*

*“There’s not enough diversity, and in the companies like Parkinson’s UK, Parkinson’s nurses.” (P2)*

Participants mentioned lack of culturally appropriate care options and the need for culturally sensitive options for treatment, some mentioned it was difficult for them to communicate the health problems in the beginning:

*“I asked for female physiotherapist. They said we don’t have this is the only one we have, and it was difficult for me to communicate with him. […] but still I found it difficult for me. I ask, I always ask, there is female, I prefer female, I feel more open to them, I can tell them everything but with men I have to think about yes or no. That was at the beginning with the first of three years. Now I’m over these things. It’s fine. But at the beginning it was difficult. Everything was difficult for me to communicate about things and tell them about my problem. It wasn’t easy.” (P4)*

A gap in targeted services for diverse communities led to feelings of exclusion in some, deterring them from accessing the services.

*“Well, there’s nothing for us. There’s nothing for Asian diverse ethnic background in the services provided at all you know, but you know the leaflets and the nurses and or the adverts you, see? […] the posters and things like that people need to see themselves reflected in the published public campaigns when they need to see themselves reflected in the people that are treating them as well and were there’s not. There’s not enough diversity […] I mean, I understand English completely, but a lot of people in especially West Yorkshire don’t and English is a second language so there need more and more translated information resources as well. You know, I think it’s a poor show at the moment to be honest.” (P2)*

## Discussion

In this qualitative study we identified a number of barriers PwP from a wide range of ME groups reported in healthcare access in the context of the NHS, a universal healthcare system. Participants were from diverse backgrounds, particularly British Asians, including individuals of Indian, Pakistani, and Sri Lankan heritage as well as a few participants from Black African, Arab African and British Arabs, groups that have not frequently been included in such studies. The findings were grouped into four main themes: Awareness and Acceptance of Symptoms in the ME Community; Socio-cultural expectations and impacts; Access to Information on Parkinson’s and Services and Experiences of Healthcare.

An interplay of low awareness and acceptance together with limited health literacy such as a lack of understanding of Parkinson’s non-motor symptoms or awareness of available healthcare services serve as significant barriers to healthcare access in ME PwP. These challenges are further compounded by the stigma associated with the condition, socio-cultural expectations like cultural stoicism and limited access to necessary resources.

In the first theme participants across ethnicities frequently failed to recognise early symptoms as signs of a severe condition, which led to delayed healthcare seeking and potentially exacerbating symptoms due to missed early intervention opportunities^9^. The majority of our participants reported having been unaware of PD or mistakenly attributing its symptoms to other conditions before diagnosis. These findings are in line with previous reviews^2,5^, which found a lack of awareness of PD also in other populations in developed countries, suggesting this is a widespread issue, not limited to people from ME backgrounds. However, low health literacy regarding the symptoms of Parkinson’s appears to be particularly common in PwP from ME groups and previous studies have shown that PwP from ME groups are diagnosed later than PwP from other backgrounds^10^. This lack of awareness can lead to delayed diagnosis and treatment because individuals may only seek medical attention for the more noticeable motor symptoms, perhaps dismissing the full spectrum of the disease.

We also found that PwP from ME groups frequently did not connect non-motor symptoms such as urinary or sleep disturbances with the disease itself and furthermore had difficulty in reporting non-motor symptoms. They often reported considerable stigma associated with these, particularly for mental health symptoms such as anxiety or depression. This finding is similar to previous studies reporting that Black Americans are likely to self‐report less disability relative to clinician‐observed motor impairment and are at higher risk of delayed or missed PD diagnosis^11^. Many participants in our cohort, especially those who were from Asian backgrounds, felt embarrassed and preferred to keep symptoms like incontinence and constipation to themselves to avoid judgement. It has been reported that some individuals from Asian and Black backgrounds have more marked non-motor symptoms, including heightened pain experience^4,12^. However, our study found that the majority are unaware of these non-motor symptoms and are reluctant to discuss complicating their healthcare treatment. Similarly, Hurt et al. ^13^ reported that those with lower health literacy are less likely to report non-motor symptoms to their healthcare professionals, with up to 72% of PwP not reporting non-motor symptoms. This low health literacy regarding non-motor symptoms may hinder early intervention and disease management, e.g. through high rates of missed appointments in people from ethnic minorities. For example, in people with chronic pain in PD^4^, significant racial disparities have been reported in the treatment of similar populations as those interviewed in our study (Black and Asian participants) and white patients with PD. These were more likely to receive appropriate pain relief compared to Black and Asian patients, with 83% of white patients receiving pain relief compared to only 48% of Black patients and 43% of Asian patients.

Stigma emerged as a separate and significant barrier together with cultural barriers that made communicating health issues like non-motor symptoms, especially sexual health, anxiety, constipation, and urinary incontinence more challenging. Participants also had privacy concerns and feelings of shame and embarrassment around discussing these non-motor symptoms. In our study this was observed in those from Asian and Arab cultures, especially in the Muslim participants from both South Asian and Arab backgrounds, as well as individuals from other religious backgrounds, like Christians, Hindus, and Sikhs, who exhibited a marked reluctance to discuss intimate issues unless directly questioned. This indicates the need for a significant degree of sensitivity when engaging participants from ME backgrounds to encourage meaningful conversations. Participants reported particularly strong stigma around mental health symptoms, which discouraged seeking support. Stigma surrounding mental health and chronic illnesses, including PD, is prevalent among minority groups in the UK where cultural perceptions can exacerbate feelings of shame and reluctance to engage with healthcare services^14–16^. Such stigma surrounding PD symptoms, particularly non-motor symptoms, can lead to social isolation^17^. Emphasizing empathy and compassionate communication (see Table 1) can help clinicians normalize conversations around these symptoms and reduce the associated stigma.

**Table 1:** Strategies to help Addressing Healthcare Barriers Faced by PwP from ethnic minority groups

Barrier	Strategies
**Lack of awareness of** **PD symptoms and services**	Community outreach awareness campaigns. Using social media platforms popular within minority ethnic communities to disseminate awareness videos and information about PD symptoms.
**Stigma Around Symptoms**	Clinicians should be aware about the stigma ME-PwP may feel, especially around non-motor and mental health symptoms. Emphasizing empathy when discussing symptoms to normalize the conversation and reduce stigma.
**Embarrassment about Non-Motor Symptoms**	Using sensitive questioning techniques. Being respectful and indirect approaches to discuss personal topics, allowing patients to share without embarrassment. Introduce private, non-verbal symptom reporting tools, such as digital questionnaires in different languages to help patients disclose sensitive issues. Having private consultations by ensuring patient privacy which will encourage open discussion without fear of embarrassment.
**Feeling Unheard / Dismissed**	Using active listening to ensure that patients feel heard by acknowledging their experiences and symptoms Scheduling follow-up visits to address unresolved concerns, reinforcing that their needs are being considered.
**Lack of Compassion and Cultural Sensitivity**	Include compassionate care practices as part of training. Having a compassionate environment and care practices to improve patient interactions.
**Limited Representation** **in Workforce and Materials**	Diversity in healthcare staff to increase visible diversity in clinics.
**Language and Communication Barriers**	Culturally relevant educational materials are beneficial by using materials that represent diverse ethnicities and provide resources in multiple languages.
	Clinicians should use clear and simple straightforward language avoiding medical jargon and confirming understanding.

In our second theme we found that socio-cultural expectations like family duties impacted and often delayed healthcare-seeking behaviour, especially among South Asian women. Some participants reported cultural expectation to endure symptoms without “making a fuss” or actively seeking help, which was often described as “crack on.” This cultural stoicism valuing silence and resilience was reported particularly in reference to enduring non-motor symptoms like pain or anxiety without expressing discomfort. This can lead to a reluctance to seek medical intervention, potentially worsening symptoms and becoming a barrier to effective disease management.

Women with Parkinson’s disease often also face distinctive and deeply personal challenges around caregiving, domestic responsibilities and self-image. Many women with PD report a strong sense of obligation to continue fulfilling family and household roles despite PD-related limitations. This responsibility to maintain caregiving roles often leads to delays in seeking medical help contributing to under-treatment and adverse health outcomes. Previous studies highlighted that these cultural and gender-based expectations lead women to feel additional psychological distress, impacting self-image and increasing their vulnerability to depression and anxiety. Mathur et al. ^18^ found that over 60% of women with PD experience a negative impact on self-image, particularly due to visible symptoms like tremors and slowness, which reinforces feelings of social stigma^18^. Subramanian et al. ^19^ therefore emphasize the need for customized care that addresses both the physical and psychosocial needs of women with PD advocating for culturally sensitive resources and targeted interventions to alleviate the specific burdens women face in managing both PD and their caregiving roles^19^.

In our third theme language and communication barriers were significant obstacles faced by many PwP from ME backgrounds. Older generations often do not have English as their first language and the lack of culturally suitable information poses significant challenges. A language barrier can lower medication adherence; patients might also visit healthcare providers less often and have a lower understanding of the condition, raising the risk of medical complications^20^. It can make it difficult for individuals to communicate health issues with neurologists, understand healthcare information and utilize necessary resources. This situation leaves a sense of frustration and exclusion among participants. Many were unaware of where to seek help. There is a considerable shortage of accessible information for ME-PwP^21^ which could exacerbate the issue of low health literacy. These barriers highlight the need for improved culturally sensitive communication and information dissemination to better support PwP from ME backgrounds. Such cultural and linguistic barriers, such as language proficiency and varying beliefs about illness and treatment, have also been identified as major causes of low access to healthcare services among immigrant patients in general^22^. It has been proposed^17^ that delivering a PD diagnosis with compassion and setting the stage with hope can significantly impact patients’ well-being and acceptance of their diagnosis. Clear empathetic communication is also important to help patients feel supported and avoid feelings of isolation and alienation during the diagnostic process. The inclusion of family members, culturally tailored resources and a compassionate approach can make a difference in understanding and accepting the diagnosis^17^.

In our final theme our study revealed significant barriers in healthcare provision that participants encountered during initial diagnosis and interaction with primary healthcare workers. The fear of judgement and perceived dismissiveness from healthcare workers led to delayed diagnoses and emotional distress, which was also found in the work by Robinson et al. ^23^. This can add to misunderstandings of symptoms, an impersonal diagnostic process, increasing patient discomfort, causing growing distrust in the healthcare system, and creating an atmosphere of reluctance to seek further specialist care.

Additionally, participants described barriers to accessing healthcare such as the perception of the healthcare system being busy and not accessible. They also felt that primary healthcare providers were not specialists in PD and lacked the specialized knowledge necessary for managing PD, which led to a preference for seeing consulting specialists despite the challenges of accessing such care. Participants were worried about the healthcare system’s lack of cultural understanding, such as difficulty communicating health problems with male providers due to cultural norms and the absence of diversity in the workforce representation and healthcare materials. These factors together with a lack of awareness of PD symptoms, especially the non-motor symptoms and the tendency to hide symptoms due to embarrassment all created significant obstacles that delayed diagnosis and treatment and ultimately affected the quality of care the participants received.

Together these factors, reluctance to discuss non-motor symptoms, fear of stigmatisation, lack of awareness and understanding about PD itself and the services available with the scarcity of tailored services for these communities and increased difficulties in accessing information and services could contribute to the increased healthcare disparities that broader quantitative evidence^1,4^ suggests they experience.

To address these challenges, we propose a set of strategies that clinicians can implement to support ME-PwP in overcoming these obstacles, improving healthcare access and quality (see Table 1). Use of these as well as culturally relevant educational materials, could help clinicians to provide more tailored support for increasing awareness of PD within these communities.

This study focussed broadly on Ethnic Minority identities to capture the overall issues faced by multiple groups at this early stage in research. While this approach uncovered several cultural barriers impacting healthcare-seeking behaviours among ME-PwP, such as observed reluctance to discuss non-motor symptoms, specific religious beliefs relating to healthcare barriers were not the primary focus and may not have been fully explored. There is a need for further research to examine how diverse religious beliefs specifically shape attitudes toward healthcare-seeking behaviours within ME populations. Our sample is predominantly British Asian, with a few additional participants from other minority ethnic groups, including Arab African, mixed ethnic backgrounds, and British Arab (see Supplementary Table 2). The sample size and demographic composition are limited, which may affect the generalizability of findings to other ME subgroups. Nevertheless, whilst these findings are particularly relevant to British Asian populations but also provide valuable initial insights into the experiences of other ME groups. We recognise that there will be numerous and diverse experiences within ME populations. Therefore, more research is required in populations of specific heritage to capture these nuances and diversity. Future research should compare how healthcare experiences of ME-PwPs with non-ME PwPs in the UK to provide a comparative perspective. Future studies should compare the healthcare experiences of ME-PwP with non-ME PwP in the UK and expand to include underrepresented groups, such as African, Caribbean and other ethnic minorities, to ensure a more comprehensive understanding.

Our results highlight the challenges faced by people with Parkinson’s from ME backgrounds when accessing healthcare. It is important to address these issues to reduce disparities in healthcare, which will create a more inclusive healthcare environment and services that are more culturally sensitive, ultimately leading to better healthcare access and improved quality of life.

## Methods

Interviews were conducted with 21 PD patients from ME backgrounds. We recruited participants from Minority Ethnic backgrounds with Parkinson’s who were interested in the study. Participants were recruited through local community centres and support groups for PwP, social media and snowballing. Interviews were primarily conducted in English with some in Urdu. All Interviews were virtual sessions via Zoom. The participants ranged in age from 31 to 71 years, with a mean age of 54. Of the participants, 38% were female. For detailed demographics, see Table 1.

This study is approved by Yorkshire & The Humber - Bradford Leeds Research Ethics Committee.

Interviews were recorded and transcribed verbatim using NVivo 14. Data were analysed thematically using Braun and Clarke’s reflexive approach^24^, focusing on identifying themes across the dataset. This method involved data familiarization, coding, theme searching, reviewing and defining themes using an inductive approach. Themes were refined through consultation with co-authors and a self-reflective journal maintained by the first author to ensure rigour and acknowledge biases^25^.

## Supplementary information

Supplementary Information: Participant Demographics and Themes Identified

## Data Availability

The data generated and analysed during the current study are not available as individual privacy could be compromised.
