# Supplementary material for: The barriers to receiving health care for people with Parkinson’s from predominantly Asian backgrounds in the UK
Source: NPJ Parkinsons Dis. 2025 May 20;11:131. doi: 10.1038/s41531-025-00946-9 (PMC12092625; doi:10.1038/s41531-025-00946-9)
Supplement: Supplementary file 1 — Supplementary Information: Participant Demographics and Themes Identified [file 41531_2025_946_MOESM1_ESM.docx]

| Age (years), mean ± SD | 54 ± 10.67 |
| --- | --- |
| Sex |  |
| Female, n (%) | 8 (38) |
| Male, n (%) | 13 (62) |
| Ethnicity | |
| Asian/Asian British - Indian | 9 |
| Asian/Asian British - Pakistani | 6 |
| Asian/Asian British - Bangladeshi | 0 |
| Asian/Asian British - Chinese | 0 |
| Asian/Asian British - Other (Sri Lankan) | 1 |
| Mixed/Multiple ethnic groups - White and Black African | 1 |
| Mixed/Multiple ethnic groups - White and Black African (Arab African) | 2 |
| British Arab | 2 |
| Any other ethnic groups | 0 |
| Ethnic Origins of people | Algeria, Iraq, Morrocco, Pakistan, India, Sri Lanka, Nigeria |
| Religions | Christianity (2), Islam (11), Sikh (1), Hindu (7) |

Table 2.

Participant characteristics.

Table 3.

Themes identified and their prevalence across participants.

| Themes | Sub-themes | Prevalence Among Participants |
| --- | --- | --- |
| Awareness and Acceptance of Symptoms in the ME Community | Lack of awareness of Parkinson’s symptoms | Majority of participants |
|  | Poor understanding of the disease's progression | Some of participants |
|  | Awareness, Stigma and Embarrassment relating to Non Motor Symptoms | Majority of participants |
|  | Mental Health | Majority of participants |
| Socio-cultural expectations and impacts | Community understanding | Common among participants |
|  | Cultural expectations of family roles | Common among participants |
| Access to Information on Parkinson’s and Services | Communication barriers | Some of participants |
|  | Lack of Knowledge on Access to Services | Majority of participants |
|  | Perceived lack of available information resources on Parkinson’s | Majority of participants |
